# Assessing the Severity of Illness in Patients With Coronavirus Disease in Saudi Arabia: A Retrospective Descriptive Cross-Sectional Study

**DOI:** 10.3389/fpubh.2020.593256

**Published:** 2020-11-19

**Authors:** Abdulhadi M. Alqahtani, Ziyad S. AlMalki, Randah M. Alalweet, Saja H. Almazrou, Abdullah Salah Alanazi, Mona A. Alanazi, Abdussalam A. AlShehri, Saleh AlGhamdi

**Affiliations:** ^1^Clinical Research Department, Research Center, King Fahad Medical City, Riyadh, Saudi Arabia; ^2^Department of Clinical Pharmacy, Prince Sattam Bin Abdulaziz University, Al-Kharj, Saudi Arabia; ^3^Preventive Health - General Department of Infectious Diseases, Ministry of Health, Riyadh, Saudi Arabia; ^4^Department of Clinical Pharmacy, King Saud University, Riyadh, Saudi Arabia; ^5^Department of Clinical Pharmacy, Jouf University, Sakaka, Saudi Arabia; ^6^Medical Research Unit, Prince Mohammed Bin Abdulaziz Hospital, Riyadh, Saudi Arabia

**Keywords:** COVID-19, characteristics, severe, non-severe, Saudi Arabia

## Abstract

**Objectives:** We aimed to describe the epidemiological and clinical characteristics of patients with COVID-19 in Saudi Arabia in various severity groups.

**Methods:** Data for 485 patients were extracted from the medical records from the infectious disease center of Prince Mohammed bin Abdul Aziz Hospital in Riyadh. Patients' basic information, laboratory test results, signs and symptoms, medication prescribed, other comorbidities, and outcome data were collected and analyzed. Descriptive data were reported to examine the distribution of study variables between the severe and not severe groups.

**Results:** Of 458 included patients, 411 (89.7%) were classified as not severe, 47 (10.3%) as severe. Most (59.1%) patients were aged between 20 and 39 years. Patients with severe conditions were non-Saudi, with a chronic condition history, and tended to have more chronic conditions compared with those without severe disease. Diabetes, hypertension, and thyroid disease were significantly higher in patients with severe disease. Death was reported in only 4.26% of severe patients. Only 16 (34.04%) patients remained in the hospital in the severe group.

**Conclusions:** Severe cases were more likely to have more comorbidities, diabetes, hypertension, and thyroid disorders were most common compared with non-severe cases.

## Introduction

Coronavirus disease 2019 (COVID-19) is a new human disease. The rapid spread of this epidemic has led to increased morbidity, mortality, and economic loss worldwide (1, 2). In the last month of 2019, many cases of acute respiratory illness had been reported in Wuhan, Hubei Province, China, now known as novel coronavirus–infected pneumonia (NCIP) (3, 4). As of January 31, 2020, some 9,692 NCIP cases in China had been confirmed. Globally, cases have been reported in the most of countries (5).

COVID-19 was recognized in the tests of bronchoalveolar lavage liquid from a patient in Wuhan and was affirmed as the etiology of the NCIP. Full-genome sequencing and phylogenic investigation indicated that COVID-19 was a clade distinct from the beta coronaviruses related to human severe acute respiratory syndrome and Middle East respiratory syndrome (6).

COVID-19 has features common to the coronavirus family and was classified as having beta coronavirus 2b ancestry. Given it closely resembles bat coronaviruses, it has been hypothesized that bats were the essential source of the virus. Although the starting point of COVID-19 is as yet being researched, growing evidence suggests spread to humans occurred by means of transmission from wild animals illegally sold in the Huanan Seafood Wholesale Market (6). Huang et al. (7) had reported the first 41 cases of NCIP, in which most patients had visited this market. Patients' clinical signs included fever, non-productive cough, dyspnea, myalgia, fatigue, normal or decreased leukocyte counts, and radiographic evidence of pneumonia. Organ dysfunction [e.g., shock, acute respiratory distress syndrome (ARDS), acute cardiac injury, acute kidney injury] and death can occur in severe cases (7).

Coronavirus diseases can range from not severe to very severe and even fatal respiratory infections. A retrospective study of 1,099 patients with COVID-19 from 552 hospitals and 30 provinces in China had found that 87.9% of patients had a fever and 67.7% had a cough, whereas diarrhea (3.7%) and vomiting (5.0%) were rare. Also, significantly more patients with severe disease received mechanical ventilation (non-invasive: 32.37 vs. 0%, *P* < 0.001; invasive: 13.87 vs. 0%, *P* < 0.001) compared with non-severe cases. The study showed that the most common complications of patients admitted to hospital were pneumonia (79.1%), followed by ARDS (3.37%), and shock (1.00%) (8).

The COVID-19 spread by human-to-human transmission with the same disease severity (including oxygen saturation, respiratory rate, blood leukocyte/lymphocyte count, and chest X-ray/computed tomography manifestations) (9). Subsequently, Guan et al. (8) had reported 99 instances of NCIP from the same hospital and suggested that COVID-19 infection clustered within gatherings of people in close contact, appeared to affect older men with comorbidities, and could result in ARDS.

On March 9, 2020, the Saudi Ministry of Health (MOH) had declared 4 new patients infected with COVID-19 (10). On June 10, 2020, according to the Saudi MOH, there were 3,717 confirmed cases of COVID-19, with the total number of the active cases standing at 33,515. There were 1,693 critical cases among the total active cases (11). Be that as it may, the distinction in clinical attributes between severe and non-severe cases was not reported in Saudi Arabia. This study aims to describe the epidemiological and clinical characteristics of patients with COVID-19 in Saudi Arabia in various severity groups. Strengthening the evidence base with regard to the severity of the illness could help healthcare providers better address this vulnerable population.

## Materials and Methods

### Study Design

We conducted a descriptive, cross-sectional study of all confirmed cases of patients with COVID-19 between March 1, 2020 and May 20, 2020, adhering to STROBE (strengthening the reporting of observational studies in epidemiology) guidelines for cross-sectional studies (10).

### Study Setting

We conducted a study of all patients with COVID-19 admitted to the infectious disease center of Prince Mohammed bin Abdul Aziz hospital in Riyadh, which is a MOH hospital and is one of the major referral hospitals in Riyadh, Saudi Arabia. The hospital is located east of Riyadh, on a 10,000 m^2^ plot, in a built-up area of 105,000 m^2^, comprising 5 floors with a 500-bed capacity. The hospital is equipped with 120 beds for intensive care, 63 rooms for emergencies, 15 rooms for surgery, a pavilion for radiology, some of the top laboratories in the world with American Association for Laboratory Accreditation, and outpatient clinics (12). In preparation for the pandemic, this hospital was one of the leading hospitals designated as a COVID-19 center, and as such, patients with COVID-19 were transferred to this hospital.

### Study Population

The population included males and females of all ages with confirmed COVID-19 infection in the laboratory using real-time reverse transcriptase-polymerase chain reaction (RT-PCR) who were admitted to the hospital during the study period. Admission to the ICU was for patients with confirmed COVID-19 infection who required rapidly increasing oxygen supplementation, oxygen via high-flow nasal cannula, non-invasive positive pressure ventilation, mechanical ventilation, or vasopressors (13). Therefore, as in previous studies (8, 14), cases were classified as severe or not severe. For the present paper, no exclusion criteria were applied.

### Variable Definitions

The variable of obesity was determined according to body mass index (BMI). BMI is computed as weight (kg)/height (m^2^), and obesity is classified as BMI ≥30, as per the World Health Organization (WHO) weight classification (15). Other chronic conditions were identified according to whether the patients had had any of the following diagnosed conditions: diabetes mellitus (defined as current use of diabetic-lowering medication associated with HbA1c levels ≥7% in accordance with the recommendations from the American Diabetes Association) (16), hypertension (being previously diagnosed as having hypertension by any medical professional and taking antihypertensive medication), asthma, pneumonia, kidney disease (i.e., urinary albumin creatinine ratio ≥30 mg/g and/or estimated glomerular filtration rate <60 mL/min/1.73 m^2^), cardiovascular disease (e.g., angina, myocardial infarction, stroke, or heart failure), cancer of any type, any psychiatric disease, dyslipidemia (i.e., total cholesterol ≥200 mg/dl, triglycerides ≥150 mg/dl, low-density lipoprotein cholesterol ≥100 mg/dL, or high-density lipoprotein cholesterol ≤ 40 mg/dL in males and ≤ 50 mg/dL in females), and thyroid disease. The number of comorbidities was categorized into *none, 1*, and, ≥*2* comorbidities. The presence of any symptoms was classified as yes or no. The result of chest X-rays were classified as normal or abnormal. Required mechanical ventilation was classified as yes or no, and patient outcomes were classified into died, home isolation, recovered, discharged, stable, still positive, and still in the hospital.

The available data from the patient laboratory test results were also analyzed. These included body temperature, heart rate, respiratory rate, white blood cell count, platelet count, hemoglobin, international normalized ratio, creatinine, sodium, and chloride. Systolic and diastolic blood pressure measured on admission were collected and categorized as following: systolic BP <100, 100–119, 120–140, and >140 mmHg subgroups and diastolic BP <80, 80–89, 90–100, and >100 subgroups (17).

### Data Source

Data were collected from patients' medical records by trained medical personnel. A patient's medical record consists of data on patients' basic information (age, sex, smoking status, nationality, history of any chronic conditions), laboratory test results, signs and symptoms, medications prescribed, other comorbidities, and outcomes. A well-designed and organized checklist was used to obtain and extract information from patients' medical records.

### Statistical Analysis

The data obtained were entered and analyzed using SAS version 9.4. Descriptive data were reported as dichotomous, polychotomous, and as frequencies and percentages to examine the distribution of study variables among the severe group (transferred to ICU) and the not severe group (not transferred to ICU); the chi-squared or Fisher's exact test, as appropriate, was used to compare categorical variables between groups. Continuous variables are presented as median with interquartile range (IQR) and compared, if normally distributed, using Student's *t*-test; otherwise, the Mann–Whitney *U*-test (Wilcoxon rank-sum test) was used. No imputation was performed for all tests, and a *P*-value of < 0.05 was considered to be statistically significant.

### Ethical Considerations

This research was reviewed and approved by the Institutional Review Board at King Fahad Medical City, Riyadh, Saudi Arabia, under the IRB log number 20-156. Permissions from the MOH and hospital management were obtained to conduct this study.

## Results

From March 1, 2020 to May 20, 2020, 458 patients infected with COVID-19 were reported and included in the analysis. Characteristics and comorbid underlying conditions of the included patients are presented by severity in Table 1. Of these 458 patients, 411 (89.7%) were classified as not severe, 47 (10.2%) as severe. Patients aged between 20 and 39 years made up the majority of the study population at 59.4%, followed by those aged between 40 and 59 years at 30.6%, older than 60 at 7.4%, and younger than 20 at 2.6%; a male and non-Saudi with the predominance of 86.9 and 80.4% of the study population, respectively. Some 21.8% had a history of chronic conditions; 324 (70.7%) patients had one chronic condition and 96 (20.9%) had 2 or more. The most common types of comorbidities were obesity (BMI ≥30 Kg/m^2^) according to the international WHO criteria for BMI, accounting for 97.8% of the study population, followed by diabetes in 13.5% of the cases and hypertension contributing to 10.9% of cases.

**Table 1:** Study population baseline characteristics for patients with COVID-19 by severity of illness.

**Characteristics**	**Total patients** **(*N* = 458)**	**Non-severe** **(*N* = 411)**	**Severe** **(*N* = 47)**	***P*-value**
**Age, years**				0.381
<20	12 (2.62)	6 (1.46)	6 (12.8)	
20–39	272 (59.4)	231 (56.2)	41 (87.23)	
40–59	140 (30.57)	140 (34.06)	0 (00.00)	
>60	34 (7.42)	34 (8.27)	0 (00.00)	
**Gender**, ***n*** **(%)**				0.079
Female	60 (13.1)	50 (12.2)	10 (21.3)	
Male	398 (86.9)	361 (87.8)	37 (78.7)	
**Nationality**, ***n*** **(%)**				0.025
Non-Saudi	368 (80.35)	336 (81.8)	32 (68.1)	
Saudi	90 (19.65)	75 (18.3)	15 (31.9)	
**Smoker**	14	14.29	10	12.2
**History of chronic conditions**	98	21.83	56	13.86
**Number of chronic conditions**				<0.001
None	38 (8.3)	38 (9.3)	0 (00.00)	0.487
1	324 (70.7)	323 (78.6)	1 (2.13)	
≥2	96 (20.9)	50 (12.2)	46 (97.9)	
**Comorbidity**				
Diabetes mellitus	62 (13.6)	33 (8.1)	29 (61.7)	<0.001
Hypertension	50 (10.94)	14 (3.41)	36 (76.6)	<0.001
Asthma	16 (3.51)	12 (2.93)	4 (8.51)	0.071
Pneumonia	7 (1.53)	5 (1.22)	2 (4.26)	0.108
Cardiovascular disease	9 (1.97)	6 (1.46)	3 (6.4)	0.055
Cancer	2 (0.44)	1 (0.24)	1 (2.13)	0.195
Psychiatric disease	4 (0.88)	3 (0.73)	1 (2.13)	0.353
Obesity (BMI ≥25/≥30 kg/m^2^)	236 (57.1)	235 (63.51)	1 (2.23)	<0.001
Dyslipidemia	4 (0.88)	2 (0.49)	2 (4.26)	0.054
Thyroid disease	11 (2.41)	6 (1.47)	5 (10.64)	0.002
Kidney disease	6 (1.32)	5 (1.23)	1 (2.13)	0.482
**Any symptoms**				0.685
No	50 (11.01)	44 (10.81)	6 (12.8)	
Yes	404 (88.9)	363 (89.2)	41 (87.23)	
**The result of chest X-Ray**				0.008
Normal	398 (88.05)	363 (98.41)	35 (76.09)	
Abnormal	54 (11.9)	43 (10.6)	11 (23.91)	
**Required mechanical ventilation**				<0.001
No	413 (90.6)	404 (98.8)	9 (19.2)	
Yes	43 (9.43)	5 (1.22)	11 (80.6)	
**Patient status**				<0.001
Died	2 (0.44)	0 (00.00)	2 (4.26)	
Home isolation	201 (43.9)	183 (44.53)	18 (38.3)	
Recovered	7 (1.53)	6 (1.46)	1 (2.13)	
Discharged	11 (2.4)	7 (1.7)	4 (8.51)	
Stable, still positive	207 (45.2)	201 (48.9)	6 (12.8)	
Still in hospital	30 (6.55)	14 (3.41)	16 (34.04)	

The chi-squared test and fisher's exact test in Table 1 showed that patients with severe conditions were remarkably non-Saudi (*P* = 0.025), with a chronic condition history (*P* < 0.001), and tended to have a higher number of chronic conditions (*P* < 0.001) compared with those without severe disease The percentage of patients with diabetes, hypertension, or thyroid disease were significantly higher (*P* < 0.001, *P* < 0.001, *P* = 0.002, respectively) in the severe condition group. Significantly more patients with severe disease received mechanical ventilation (80.85 vs. 1.22%). In terms of patient outcomes, death was reported in only 4.26% of severe patients. Only 16 (34.04%) patients remained in the hospital in the severe group.

Reported signs and symptoms for the study population were compared according to age group using the chi-squared test and fisher's exact test (Table 2). For the entire study population, fever, dry cough, and dyspnea were the most common symptoms upon admission. Chest pain was found to be significantly higher among patients in the age group 60 years and older. More than 43% of patients had a systolic blood pressure of 120–140 mm Hg, and 69.8% had a diastolic blood pressure of 90–100 mm Hg. Of patients aged 60 years and older, 53.1% had systolic blood pressure of 120–140 mm Hg and 78.1 had diastolic blood pressure of 90–100 mm Hg. A higher respiratory rate was observed among patients between 20–39 and older than 60 years old (median respiratory rate, 20 [IQR 19–20] for both age groups; *P* = 0.0054).

**Table 2:** Reported signs and symptoms in patients with COVID-19 by age group.

**Signs and symptoms**	**Total patients**	**Age, years**				***P*-value**
		**<20 (%)**	**20–39 (%)**	**40–59 (%)**	**>60 (%)**	
Fever	324 (70.74)	9 (75)	196 (72.1)	101 (72.14)	18 (52.94)	0.128
Dry cough	277 (60.5)	8 (66.7)	168 (61.8)	86 (61.43)	15 (44.12)	0.236
Dyspnea	128 (27.9)	2 (16.7)	69 (25.4)	48 (34.3)	9 (26. 5)	0.213
Sore throat	62 (13.54)	1 (8.33)	39 (14.34)	15 (10.7)	7 (20.6)	0.419
Diarrhea	44 (9.61)	1 (8.33)	26 (9.56)	14 (10)	3 (8.82)	0.995
Fatigue	29 (6.33)	1 (8.33)	20 (7.4)	6 (4.3)	2 (5.9)	0.668
Vomiting	23 (5.02)	0 (00.00)	17 (6.3)	4 (2.9)	2 (5.9)	0.403
Runny nose	17 (3.71)	0 (00.00)	8 (2.9)	7 (5)	2 (5.9)	0.569
Chest pain	14 (3.06)	1 (8.33)	6 (2.21)	3 (2.14)	4 (11.8)	0.012
Abdominal pain	11 (2.4)	1 (8.33)	7 (2.6)	2 (1.43)	1 (2.94)	0.485
Myalgia	7 (1.53)	0 (00.00)	5 (1.8)	2 (1.43)	0 (00.00)	0.826
Nausea	7 (1.53)	0 (00.00)	6 (2.2)	1 (0.71)	0 (00.00)	0.539
**Systolic blood pressure, mm Hg**						<0.001
<100	15 (3.6)	0 (00.00)	0 (00.00)	13 (12.8)	2 (6.3)	
100–119	171 (41.01)	6 (54.6)	116 (42.7)	38 (37.3)	11 (34.4)	
120–140	180 (43.17)	3 (27.3)	135 (49.6)	25 (24.5)	17 (53.13)	
>140	51 (12.23)	2 (18.2)	21 (7.72)	26 (25.5)	2 (6.3)	
Systolic blood pressure, mm Hg	122.74–15.63	127.81–14.94	123.09–14.89	121.45–15.66	123.67–21.43	0.0521
**Diastolic blood pressure, mm Hg**						0.046
<80	86 (20.6)	3 (27.3)	58 (21.32)	19 (18.63)	6 (18.8)	
80–89	34 (8.2)	1 (9.1)	25 (9.19)	8 (7.84)	0 (00.00)	
90–100	291 (69.8)	7 (63.64)	189 (69.5)	70 (68.63)	25 (78.13)	
>100	6 (1.44)	0 (00.00)	0 (00.00)	5 (4.9)	1 (3.13)	
Diastolic blood pressure, mm Hg	75.87–10.44	79.27–8.85	75.72–10.35	76.69–10.47	71.92–11.04	0.1105
Body temperature, °C, Median (IQR)	37 (36.7–37.7)	36.8 (36.6–37.2)	36.9 (36.7–37.7)	37.2 (36.8–37.8)	36.9 (36.7–37.3)	0.1444
Heart rate, beats per minute, Median (IQR)	86 (78–99)	92 (84–101)	84 (77–99)	90 (82–99)	82 (64–96)	0.1534
Respiratory rate, breaths per minute, Median (IQR)	20 (18–20)	19 (19–20)	20 (19–20)	19 (18–20)	20 (19–20)	0.0054

According to Student's *t*-test and the Mann–Whitney *U*-test, there were numerous significant differences in laboratory findings between patients in the severe and non-severe groups (Table 3). Patients with severe disease showed increased white blood cells (median 8.4 [IQR 6–12.3] × 10^9^/L vs. 6 [IQR 4.8–7.7] × 10^9^/L; *P* < 0.001), and platelet counts (median 244 [IQR 198–342] × 10^9^/L vs. 226 [IQR 180–277] × 10^9^/L; *P* = 0.046). Most patients in the severe group had reduced hemoglobin levels (median concentration 136 g/L [IQR 126–147] vs. 150 g/L [IQR 140–161]; *P* < 0.001) and higher international normalized ratio (median 1.03 [IQR 0.99–1.14] vs. 1 [IQR 0.97–1.05]; *P* = 0.004).

**Table 3:** Laboratory findings of patients with COVID-19 by the severity of illness.

**Laboratory findings**	**Total**			**Non-severe**			**Severe**			***P*-value**
	***N***	**Median**	**IQR**	***N***	**Median**	**IQR**	***N***	**Median**	**IQR**	
White blood cell count (WBC), × 10^9^/L	335	6.2	4.9–8	288	6	4.8–7.7	47	8.4	6–12.3	<0.0001
Platelet count (PLT), × 10^9^/L	332	229.5	182.5–284.5	285	226	180–277	47	244	198–342	0.0466
Hemoglobin (Hgb), g/dl	334	148.5	136–159	287	150	140–161	47	136	126–147	<0.0001
Hematocrit (Hct), L/L	333	0.454	0.421–0.484	286	0.459	0.428–0.487	47	0.428	0.394–0.453	<0.0001
Prothrombin time (PTT), S	235	35.1	32.1–38.1	188	34.9	31.9–37.75	47	37.2	34–43.3	0.0123
International Normalized Ratio (INR)	257	1.01	0.97–1.07	210	1	0.97–1.05	47	1.03	0.99–1.14	0.0044
Creatinine, μmol/L	79	80.8	64.9–89.6	70	81.3	66.8–89.6	9	63.4	62.3–70	0.1306
Sodium (Na), mmol/L	306	138	136–139	263	138	136–139	43	137	134–139	0.0505
Chloride (Cl), mmol/L	319	104	101–106	272	104	102–106	47	103	100–106	0.0525

Antibacterial drugs, antipyretic drugs, antimalaria drugs, antithrombotic drugs, and antiviral treatment drugs were the main treatments prescribed to patients with COVID-19. Treatment for non-severe and severe cases is shown in Table 4. Antibacterial drugs were the most commonly used class overall. A higher percentage of patients with severe conditions were treated with antibacterial drugs compared with those without (95.7 vs. 54.3%). Antimalarial drugs were the second most common type of antibiotic administered for severe cases.

**Table 4:** Treatments of patients with COVID-19.

**Laboratory findings**	**Total patients using** **a treatment**	**Non-severe** **(*N* = 329)**	**Severe** **(*N* = 122)**
Antimalarial drugs	92 (20.3)	54 (16.4)	38 (31.15)
Antiviral drugs	10 (2.1)	10 (3.04)	0 (00.00)
Antibacterial drugs	153 (33.9)	108 (32.83)	45 (36.9)
Antithrombotic drugs	91 (20.2)	70 (21.3)	21 (17.2)
Antipyretic drugs	105 (23.3)	87 (26.4)	18 (14.8)

## Discussion

COVID-19 is currently a major infectious disease, causing substantial morbidity and mortality worldwide. A better understanding of patient characteristics in terms of illness severity could lead to increased adoption of appropriate care in this group, consequently reducing the burden of infection. In this retrospective study, a total of 458 patients with COVID-19 were included, of whom 47 were recognized as having severe disease. The overall mortality rate was 0.44% by May 20, 2020, all in the severe group. Most of the patients were in stable condition or were discharged from the hospital, suggesting that the COVID-19 had been controlled and treated effectively.

Few systematic reviews and meta-analyses have been conducted to describe the clinical characteristics of COVID-19 patients in China (18–22). Among those reviews, Cao et al. (21) and Hu et al. (22) were the most inclusive, with a total of 46,959 and 47,344 patients, respectively. These reviews included all published retrospective cohort studies and case series until March 10, 2020. The mean age in the Cao et al. review was 46 years, whereas Hu et al. had reported an average age older than 40 years. In both reviews, there was an equal distribution of both sexes. The most frequently reported clinical manifestations in both reviews were fever (85%) and cough (65%), followed by dyspnea (38%) (21) and fatigue (42%) (22). Cao et al. had found that hypertension and diabetes were the most commonly reported comorbidities, accounting for 18 and 10%, respectively (21); similar figures had been reported by Hu et al. (22).

Lechien et al. had described the clinical characteristics of patients with non-severe COVID-19 from multiple European countries (23). This retrospective study included 1,420 patients (mean age 39, 67% female). The most reported symptoms were headache and loss of smell, accounting for 70% of all cases; only 45% of patients reported fever. As for comorbidities, 9% of the patients had hypertension. This study reported differences in clinical presentation between males and females and among different age groups. For example, female patients tended to have headaches and loss of smell, whereas male patients tended to have fever and cough. As for age, younger patients had ear, nose, and throat complaints, whereas older patients reported having fever and fatigue.

We found few similarities between meta-analysis findings (21, 22) and our findings in terms of mean age, and the most commonly reported clinical manifestation. In our study, however, the male to female ratio was higher, and fever and cough were also commonly reported, which is in line with the findings of Lechien et al. (23). The proportion of patients with diabetes among the severe cases was significantly higher than among the non-severe. Likewise, the proportion of patients with hypertension tended to be higher among the severe cases than in the severe group. Evidence from multiple meta-analyses suggests that hypertension and diabetes have been strongly associated with disease severity and poor prognosis (19, 24–26). In Saudi Arabia, 28% of the population has been considered obese (27). Hypertension and diabetes have also been found to affect 15 and 24% of the Saudi population, respectively (28–30). The prevalence of such conditions is expected to be even higher due to obesity and a sedentary lifestyle (31–33). These characteristics can lead to longer hospitalization and ICU admission, which increases the burden on the healthcare system.

One of the worth noting findings in this study, which was not previously been reported elsewhere, is the number of expatriates, which represents 80% of the samples. A possible reason for this high percentage is low economic status. In a study conducted by Al Khamis et al. (34) two-thirds of expatriates have low incomes (less than SR2000 per month). Al Khamis et al. have also reported that 65% of expatriates are married but without their families (34). Despite the majority of non-Saudis not having severe disease, the majority live in shared accommodations and worker housing units, where infection spread is expected to be higher. In addition, the MOH has started an “active screening” program, also called “active surveillance,” in overpopulated areas, which are typically inhabited by expatriates, for early detection of COVID-19 cases; this program could explain the higher prevalence among expatriates (35).

To our knowledge, this study is one of few studies that describes the characteristics of patients with COVID-19 in Saudi Arabia. In our study, we collected all available variables for patients to enable a clear picture of the characteristics of patients with COVID-19 in terms of severity of illness in the Riyadh city population. In terms of symptoms, in our sample, roughly 11% of patients were asymptomatic. Estimating the asymptomatic percentage of COVID-19 patients is an essential measurement for decision makers. Despite the strengths of our study, it also has limitations. First, this study was performed in one hospital in Riyadh; thus, we cannot generalize these results to the entire COVID-19 population in Riyadh. Moreover, the sample size was 458 patients, which could have led to some non-significant differences in the subgroup analysis because the power of the study was limited by sample size. Finally, because the laboratory tests are only performed upon request by the physician according to the patient's condition, we have observed that some test results have a very high percentage of missing values.

In this study, People with Diabetes, hypertension, and thyroid disease were at higher risk of severe disease requiring hospitalization and subsequently ICU admission. Therefore, our study results could help both healthcare providers and decision makers during this pandemic. For healthcare providers, these results can help them identify those high-risk patients and provide them with early intervention to prevent complications. Additionally, patients at high risk should be targeted for screening in order to diagnose them as soon as possible to start providing care. Not only healthcare providers but also decision makers might be able to use the results of this study to apply procedures to reduce the outbreak of this virus and other infections. This can be achieved by enforcing workplace policies which prohibit the physical attendance for people at higher risk of infection and arrange for distant working schedule where appropriate. Additionally, hospital outpatient follow-up visits for people with higher risk should be performed using Teleclinics and or phone calls where appropriate. Medication refills and collection also need be arranged with a courier. Applying these measures could potentially reduce the spread of infection to people at high risk and subsequently reduce admission and future complication.

According to our study, ~80% of patients with COVID-19 patients were non-Saudi, which might be associated with living in overpopulated areas. According to these study results, the government should adopt new regulations to reduce overpopulated residences. By adopting these regulations, reducing the spread of infection in these places not only helps reduce the burden during the COVID-19 pandemic but also during all possible future infectious diseases.

## Data Availability Statement

The raw data supporting the conclusions of this article will be made available by the authors after obtaining approval from the local ethical committee, without undue reservations.

## Author Contributions

AMA: study design, data collection, and writing. ZA: study design, data analysis, and writing. RA: data collection and writing. SHA and ASA: manuscript drafting. MA and AAA: data collection. SA: writing and critical revision of the manuscript. All authors approval of the final version.

## Conflict of Interest

The authors declare that the research was conducted in the absence of any commercial or financial relationships that could be construed as a potential conflict of interest.
